# Doppler Ultrasonography Derived Maximal Systolic Acceleration: Value Determination With Artificially Induced Stenosis

**DOI:** 10.1177/15385744221076269

**Published:** 2022-03-02

**Authors:** Jeroen J. W. M. Brouwers, Louk P. van Doorn, Laurie Pronk, Rob C. van Wissen, Hein Putter, Abbey Schepers, Jaap F. Hamming

**Affiliations:** 1 Department of Vascular Surgery, 4501 Leiden University Medical Center, Leiden, The Netherlands; 2 Department of Surgery, HagaHospital, The Hague, The Netherlands; 3 Department of Medical Statistics and Bioinformatics, 4501 Leiden University Medical Center, Leiden, The Netherlands

**Keywords:** doppler duplex ultrasound, in vivo techniques, peripheral arterial disease, medial calcific sclerosis

## Abstract

In diagnosing peripheral arterial disease (PAD), medial arterial calcification (MAC) hampers arterial compression and could lead to unreliable ankle brachial index (ABI), toe brachial index (TBI) and toe pressure (TP). Doppler ultrasonography (DUS) derived maximal systolic acceleration (ACCmax) might be more accurate to diagnose PAD. In an in vitro study, a strong correlation between ACCmax and the severity of stenotic disease was determined. The aim of this study was to investigate the ACCmax in correlation with conventional non-invasive diagnostics in an in vivo setting. **Methods:** In twelve healthy individuals, an arterial stenosis was mimicked by compression on the common femoral artery by an ultrasounds probe, creating a local stenosis of 50%, 70% and 90%. The ABI, TBI, TP and several DUS parameters (including ACCmax) were assessed at the ankle during these different degrees of stenosis. All DUS parameters were measured separately by two observers to determine the interobserver variability. **Results:** Overall the ABI, TBI, TP, ACCmax, ACCsys and PSV decreased significantly when the degree of stenosis increased. The ACCmax showed the highest correlation with the degree of stenosis (r −.884), compared to ABI (r −.726), TBI (r −.716) and TP (r −.758). Furthermore, the interobserver variability of ACCmax was excellent, with an intraclass correlation coefficient (ICC) of .97. **Conclusion:** ACCmax is an accurate non-invasive DUS parameter to diagnose and assess the severity of a mimicked arterial stenosis in healthy individuals. Further prospective assessment of the clinical value of ACCmax and its potential benefits in patients with PAD is needed.

## Introduction

The severity of peripheral arterial disease (PAD) is primarily assessed by the ankle brachial index (ABI), toe brachial index (TBI) and toe pressure (TP).^1^ However, due to incompressible arteries in patients with medial arterial calcification (MAC), the ABI, TBI and TP can be falsely elevated leading to unreliable results.^2-5^ MAC is mostly seen in patients with diabetes mellitus (DM), chronic kidney failure and elderly patients.^6^ In these patients, ABI, TBI and TP will therefore not provide an adequate estimation of the blood flow to foot and toes.^7,8^ The prevalence of PAD in people with DM is 20–30%,^9^ and increases to 65% in patients with diabetic foot ulcer (DFU).^10^ MAC can be present in up to a third of patients with DM,^11^ and in patients with critical limb ischemia (CLI) circa 20% have incompressible arteries.^12^ For these patients, an alternative non-invasive accurate diagnostic parameter is needed to assess the severity of PAD.

Two recent reviews showed the poor results and insufficient evidence of bedside tests for diagnosing PAD among patients with DM. These authors advocated for more studies and an alternative diagnostic technique.^7,8^ A relatively new Doppler ultrasonography (DUS) parameter, maximal systolic acceleration (ACCmax), can be used in detecting PAD and better estimating its severity independently of blood pressure measurements.^5,13,14^ It measures the acceleration of blood flow by quantifying the maximal slope of the systolic doppler curve. Recently, our in vitro study showed that the ACCmax decreased as the severity of stenosis increased. Also, a strong correlation was found between the ACCmax and the intra-arterial pressure gradient.^13^ ACCmax has potentially important benefits compared to the conventional non-invasive bedside tests regarding the influence of incompressible arteries.^5,8,13-15^

To further investigate the value of ACCmax in PAD, an in vivo study was conducted with artificially created arterial stenosis in healthy individuals. The aim of this study was to compare the ACCmax with conventional non-invasive arterial pressure measurements and other DUS parameters to determine the severity of arterial stenosis.

## Materials and Methods

### Ethical Considerations

This study follows the declaration of Helsinki. The medical ethical committee of the tertiary academic hospital granted permission to perform this study (P16.251). Participation was voluntarily and without obligation. All the study participants received a clear letter of information and signed informed consent.

### Design

In this prospective in vivo study, the study population consists of healthy male participants between 18 and 30 years old with a normal circulation. Subjects were not included when suffering from PAD, DM, cardiac disease, or other vascular diseases (among other things Raynaud’s phenomenon or vasculitis).

**Figure 1.**
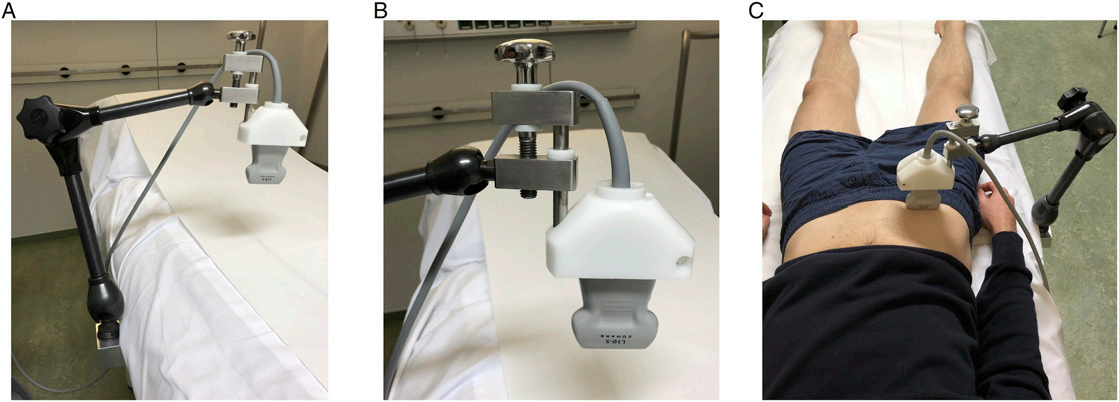
Overview of the test setup. A: the adjusting arm is displayed. B: the ultrasound transducer was attached to the white holder of the adjusting arm. By the screw construction it was possible to obtain compression on the CFA. C: An overview of the experimental test setup is displayed.

In this study, a developed test setup was used, as shown in figure 1. The instrumental affairs department of the academic hospital made a robust adjusting arm, which was attached to the examination bed (figure 1(a)). On the other side of the arm, an ultrasound transducer was attached to the white holder (figure 1(b)), a Z-One Zonare duplex device with a transducer L 10-5 was used. Due to the adjustable screw construction (figure 1(b)), the ultrasound transducer was able to acquire well-balanced compression on the common femoral artery (CFA), creating a modifiable stenosis in the CFA. Because an ultrasound transducer was used to get compression on the CFA, the degree of stenosis was directly obtained by duplex as reference test. The degree of compression can be adjusted using the screw construction, adjusting the height and thereby the extent of compression of the ultrasound transducer. Hence, the transducer that was connected to the arm was bifunctional: causing and also directly showing the degree of stenosis. Figure 1(c) shows an overview of the test setup. In our test setup, 2 DUS devices were used: one causing and measuring the degree of stenosis in the CFA, the other device measuring the DUS parameters (including the ACCmax) at the posterior tibial artery. Three degrees of stenosis were obtained: 50%, 70% and 90% by compression on the CFA. During these measurements, there was a continuous monitoring of the obtained degree of stenosis in the CFA. After about 30 seconds, to confirm the stenosis was stable, the following measurements were obtained at the posterior tibial artery: the ABI, TBI, TP, ACCmax, mean systolic acceleration (ACCsys), acceleration time (AT) and peak systolic velocity (PSV). Due to the method of local compression on the CFA, the artery disformed into an oval shape instead of a concentric stenosis, therefore reduction in cross-sectional area was used to determine the degree of stenosis, instead of the diameter reduction measurement.

In case of emergency, the modified arm could be simply removed by a separate screw construction, this procedure was not necessary during the study.

### Doppler Ultrasonography

**Figure 2.**
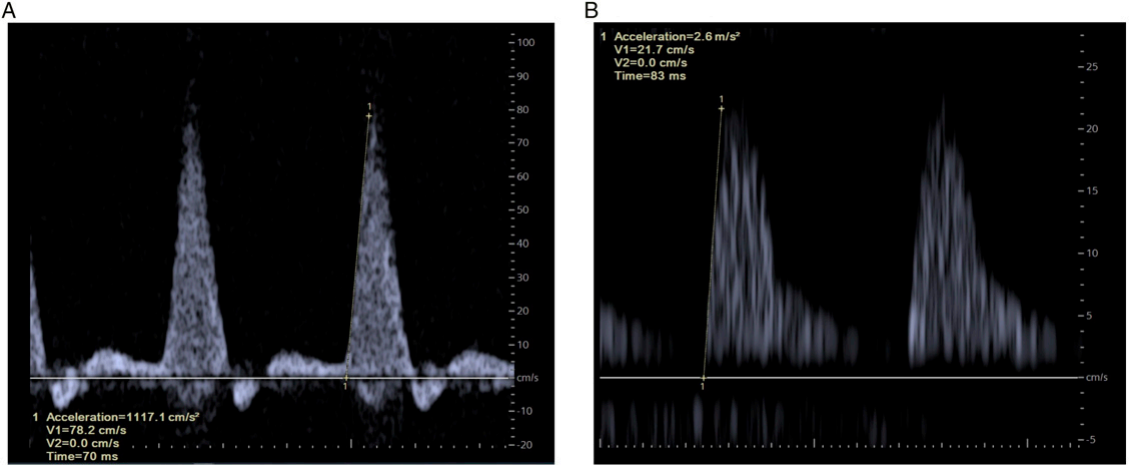
Doppler waveforms. A: a normal waveform is shown without the presence of peripheral arterial disease (ACCmax 11.2 m/sec^2^). B: a post stenotic signal in the tibialis posterior artery is obtained with a decreased ACCmax (2.6 m/sec^2^). In both figures, the ACCmax is measured at the maximal slope in the systolic phase. Note the differences in scales between the figures.

All DUS measurements were done by two separate investigators, using an Acuson S2000 System, Helix Evolution (Siemens Medical Solutions, Ultrasound Division, Issaquah) equipped with a 9L4 9-4 MHz linear transducer. All measurements were performed with a fixed 60-degree angle of insonation. To determine the interobserver variability, the two investigators were unaware of the measurements of each other. However, the investigators were aware of the degree of stenosis. The ACCmax was calculated by a computer at a single representative curve, as described in Brouwers et al.^13^ The ACCmax occurs at the maximal slope in the systolic phase and is expressed in m/sec.^2^ Figure 2 shows an example of a normal and a divergent waveform including ACCmax measurements. No additional software is necessary to obtain the ACCmax. By clicking on two points in the screen, there will be one tangent line. This tangent line must be placed manually at the maximal slope in the systolic phase. The computer automatically calculates the acceleration of the tangent line in m/sec^2^ (= maximal systolic acceleration). The ACCmax is always measured distal to the stenosis (for example, at the distal posterior tibial artery). In an in vitro study, it is suggesting that the ACCmax does not depend on the distance between the stenosis and the measurement point.^13^ ACCmax should not be confused with either acceleration time (AT) or mean systolic acceleration (ACCsys). ACCsys is the slope between the beginning of the systolic upstroke and the peak of systole and is calculated using the following equation: ACC_sys_ = ΔV_sys_/AT.

### Statistical Analysis

The power analysis of this study was based on a paper of Julious et al,^16^ which describes that for an explorative study looking at means and standard deviations, 12 test subjects are required. All statistical analyses were performed using SPSS statistics 25.0 software ® (IBM, Armonk, NY). Mixed model analysis was used to determine if there are overall differences between a parameter at multiple test setups (degree of stenosis). A Bonferroni correction was applied for each parameter to correct for multiple testing of different test setups (no stenosis vs 50% stenosis, 50% stenosis vs 70% stenosis, and 70% stenosis vs 90% stenosis). Differences with P < .05 were considered statistically significant. Following a bivariate correlation analysis, the correlation between parameters was calculated by Pearson’s r. A Pearson’s r of >70 is considered a high correlation, .50–.70 refers to a moderate correlation. The interobserver variability was assessed using an intraclass correlation coefficient (ICC). An ICC of >.90 indicates an excellent agreement between the different observers.

## Results

Twelve healthy subjects participated in the present study, without any dropouts. The following target test setups were applied to the test subjects: no stenosis, 50%, 70% and 90% degree of stenosis. Looking at the degree of stenosis, as shown in Table 1, a corresponding stenosis degree was created using compression by the inguinal ultrasound transducer on the CFA. These actual test setups were: no stenosis, 50% (±1.8), 70% (±1.1) and 89% (±1.2), which were reliable values for this investigation. All tests were well tolerated by the subjects; there were no obvious pain complaints. Subjects did experience temporary discomfort by pressure in the groin, and a pins and needles sensation in the leg at high grade stenosis.

**Table 1.** Overview of Different Test Setups.

Target Test Setups	Mean Actual Degree of Stenosis
no stenosis	No stenosis
50%	50% (±1.8)
70%	70% (±1.1)
90%	89% (±1.2)

Abbreviations: The standard deviation (±SD) is given in percent. The degree of stenosis is given in reduction in cross-sectional area.

Table 2 shows an overview of the assessed parameters at different test setups. Upon increasing degrees of CFA stenosis, overall a significant reduction was seen in ABI (P < .001), TBI (P < .001), TP (P < .001), ACCmax (P < .001), ACCsys (P < .001) and PSV (P<.001). Furthermore, AT was significantly increasing (P < .001) when increasing the degree of stenosis.

**Table 2.** Overview of Assessed Parameters.

	No Stenosis	50% Stenosis	70% Stenosis	90% Stenosis
ABI	1.1 (±.11)	.99 (±.14)	.89 (±.15)	.59 (±.22)
TBI	.93 (±.15)	.86 (±.16)	.75 (±.14)	.51 (±.16)
TP	122 (±18)	113 (±21)	98 (±14)	65 (±16)
ACCmax	8.6 (±.9)	7.5 (±2.5)	4.6 (±1.0)	1.0 (±.5)
ACCsys	6.5 (±1.1)	5.6 (±1.7)	3.9 (±1.1)	.9 (±.3)
AT	85 (±13)	80 (±16)	91 (±17)	116 (±27)
PSV	52 (±12)	45 (±16)	33 (±8.3)	15 (±13)

Abbreviations: The mean ankle brachial index (ABI), toe brachial index (TBI), toe pressure (TP) in mm Hg, maximal systolic acceleration (ACCmax) in m/sec^2^, mean systolic acceleration (ACCsys) in m/sec^2^, acceleration time (AT) in in milliseconds and peak systolic velocity (PSV) in cm/sec are given for the different test setups, including the standard deviation (SD). The degree of stenosis is given in cross-sectional area reduction.

**Figure 3.**
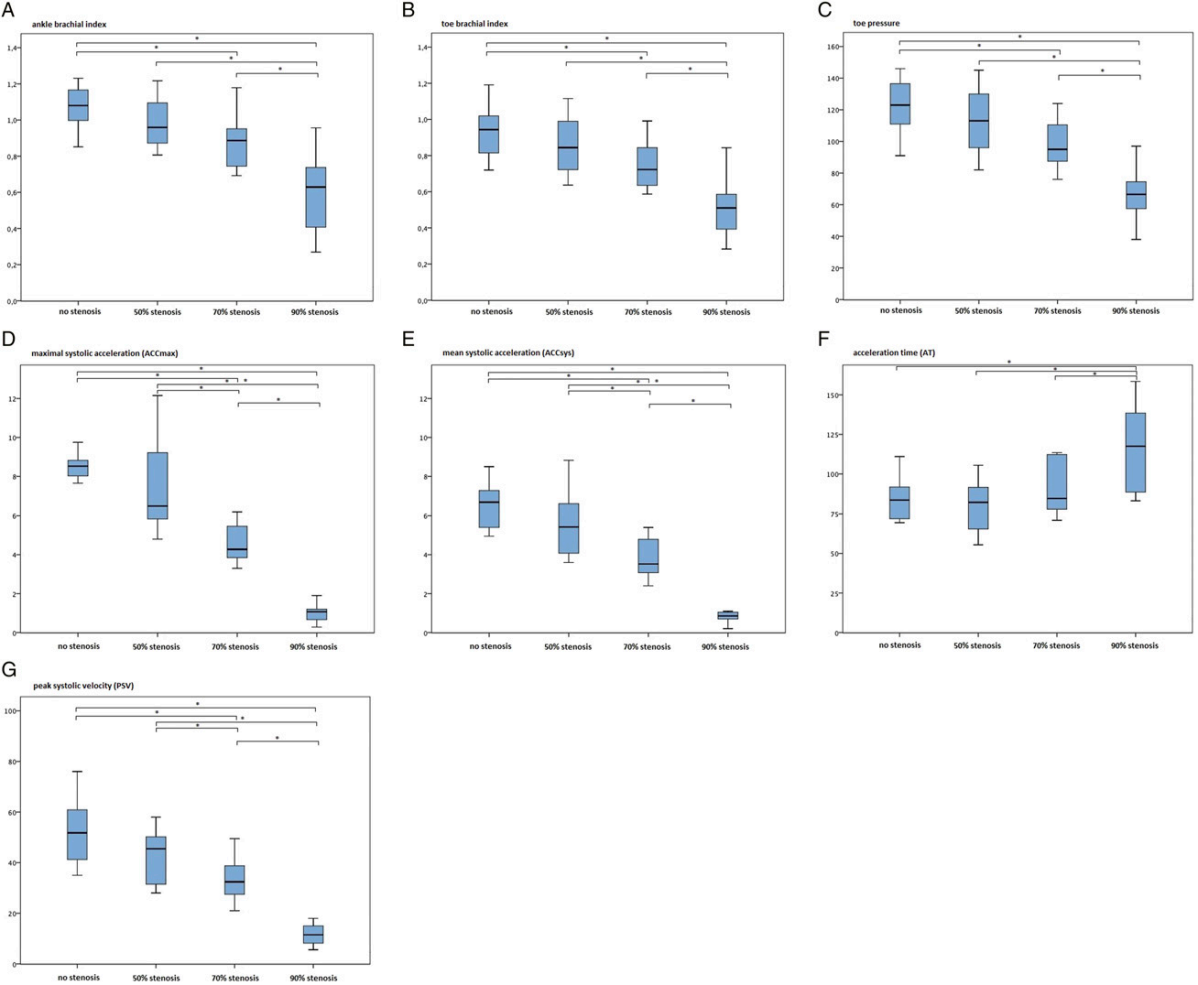
Boxplots of diagnostic parameters for different test setups. A: ankle brachial index (ABI), B: toe brachial index (TBI), C: toe pressure (TP) in mm Hg, D: maximal systolic acceleration (ACCmax) in m/sec^2^, E: mean systolic acceleration (ACCsys) in m/sec^2^, F: acceleration time (AT) in milliseconds and G: peak systolic velocity (PSV) in cm/sec. The boxplots are representing the median, 25% quantile, 75% quantile and 1.5 interquartile range (top and bottom whiskers) per test setup. Statistically significant differences (P < .05) between test setups are marked with *.

Figure 3 depicts boxplots for all parameters, the Bonferroni adjusted P-values were determined between every test setup (no stenosis vs 50% stenosis, 50% stenosis vs 70% stenosis, and 70% stenosis vs 90% stenosis): for ABI P = .308, P = .330, P < .001*; for TBI P = .615, P = .094, P < .001*; TP P = .763, P = .100, P < .001*; for ACCmax P = .287, P < .001*, P < .001*; for ACCsys P = .177, P = .001*, P < .001*; for AT P = 1.000 P = .422, P = .001*; and for PSV P = .203, P = .003*, P < .001*, respectively.

The correlation coefficient between parameters and also with the degree of stenosis is shown in Table 3. In our analysis, the ACCmax had the best correlation (r −.884) to the degree of stenosis, followed by ACCsys (r −.861), TP (r −.758), PSV (r −.741), ABI (r −.726), TBI (r −.716) and AT (r .503). The ACCmax was also highly correlated to ACCsys (r .969), ABI (r .782) and TP (r .743).

**Table 3.** Overview of the Correlation Coefficients Between the Degree of Stenosis and Parameters.

Correlation Analysis		Pearson Correlation Coefficient r (all with P-Value <.01)
ACCmax	Stenosis degree	**−.884**
	ABI	.782
	TBI	.697
	TP	.743
	ACCsys	.969
	AT	−.602
	PSV	.797
ABI	Stenosis degree	**−.726**
	TBI	.850
	TP	.809
	PSV	.554
	ACCsys	.782
	AT	−.684
TBI	Stenosis degree	**−.716**
	TP	.954
	PSV	.628
	ACCsys	.709
	AT	−.464
TP	Stenosis degree	**−.758**
	PSV	.719
	ACCsys	.762
	AT	−.448
ACCsys	Stenosis degree	**−.861**
	PSV	.804
	AT	−.606
AT	Stenosis degree	**.503**
	PSV	−.304
PSV	Stenosis degree	**−.741**

Abbreviations: Ankle brachial index (ABI), toe brachial index (TBI), toe pressure (TP), maximal systolic acceleration (ACCmax), mean systolic acceleration (ACCsys), acceleration time (AT) and peak systolic velocity (PSV).

### Interobserver Variability

All DUS parameters were measured by two independent investigators to obtain the interobserver variability in this in vivo study. As shown in Table 4, the intra class correlation coefficient (ICC) was .97 for ACCmax. Also, PSV had an excellent agreement in the measurements with an ICC of .91. ACCsys and AT had a good level of agreement, respectively, an ICC of .71 and .72.

**Table 4.** The Interobserver Variability for Different DUS Parameters.

	Intra Class Correlation Coefficient
ACCmax	.97
ACCsys	.71
AT	.72
PSV	.91

Abbreviations: Maximal systolic acceleration (ACCmax), mean systolic acceleration (ACCsys), acceleration time (AT) and peak systolic velocity (PSV).

## Discussion

In the present study, PAD was mimicked in an in vivo setting by controlled local compression on the common femoral artery in healthy study participants. A strong correlation was found between ACCmax and the degree of an artificially introduced stenosis. The ACCmax proved to be superior to ABI, TBI, TP, ACCsys and PSV and had an excellent interobserver variability. Therefore, ACCmax measurement is a promising reliable non-invasive tool to assess the severity of PAD.

Previous studies indicated that the ACCmax can be used as an accurate PAD diagnostic marker, even in patients with DM.^5,13,14^ Similar to our in vitro study,^13^ in this in vivo study a strong correlation between the ACCmax and the degree of stenosis was demonstrated (r −.884). In addition, our previous in vitro study showed a good correlation between the ACCmax and the intra-arterial pressure gradient (R^2^ .937). A high ACCmax value precludes a hemodynamic inflow problem and excludes the presence of PAD proximal of its measuring point. In the present study, in a hemodynamically significant stenosis of 70% reduction in cross-sectional area, a mean ACCmax of 4.6 m/sec^2^ was found. This is in accordance with the previous results of our group indicating that a high ACCmax (>10 m/s^2^) can exclude the presence of PAD with a negative predictive value of 95%. An ACCmax of below 6.5 m/s^2^ is strongly indicative of PAD with a positive predictive value of 99%. In that paper, it is concluded that the ACCmax is an accurate marker that could offer significant benefits for the diagnosis of PAD, especially in DM.^5^ Buschmann et al revealed a threshold of 5.0 m/s^2^ for diagnosing PAD (based on digital subtraction angiography), and showed a better ACCmax area under the curve compared to ABI and relative pulse slope index in patients with and without DM.^14^ So, ACCmax appears to be more accurate in detecting PAD than the conventional non-invasive pressure measurements. Additionally, there are some practical advantages of using ACCmax. It can be measured at any point in the artery, hence avoiding effects associated to a local calcified plaque. Furthermore, ACCmax measurements can be obtained in a very short time (data acquisition time of less than 1 minute), in contrast to ABI, TBI and TP (more than 10 minutes).^14^

There are several considerations to be made in favour of ACCmax measurements. ABI, TBI and TP are prone to be falsely normal or elevated due to incompressible peripheral arteries, especially in elderly patients that have a history of DM or have suffered from renal disease for a longer period of time, resulting in medial arterial calcification (MAC).^6,14,17-19^ ABI and TBI have a rather low sensitivity for diagnosing PAD in patients with DM, with a sensitivity of 45% and 64%, respectively.^20^ In addition, slightly higher numbers have been reported on the sensitivity and specificity of TP for diagnosing PAD in patients with DM, respectively, 74% and 72%.^21^ In contrast to external blood pressure measurements (ABI, TBI and TP), DUS measurements circumvent this limitation regarding MAC.^8^ By measuring ACCmax, there is no external blood pressure measurement that can be influenced by vessel stiffness. Sung et al showed no influence of vessel compliance (as in vessels with MAC) on the changes in peak systolic velocity (PSV), acceleration time (AT), or acceleration index (AI),^15^ suggesting ACCmax is also not affected by vessel compliance changes. In clinical studies, there has been concluded ACCmax can be used to diagnose PAD accurately in patient with high risk of MAC.^5,14^ Therefore, the ACCmax is also a potential accurate measurement of perfusion in patients with DM, independently of presence of MAC.

With respect to the reproducibility, a wide range of results was published for ABI.^22-25^ De Graaff et al showed an interobserver variability (ICC) for ABI of .92 and for TP of .88 at the same day. Moreover, a 1-week interobserver repeatability coefficient of 27% and 41 mm Hg for ABI and TP were found, respectively.^25^ In accordance with the results of the previous in vitro study,^13^ an excellent agreement for ACCmax was revealed in the present study, ICC .99 and .97, respectively. Since these studies were experimental (in vitro and in vivo) care must be taken when comparing it to the results of de Graaff et al.

The highest clinical value of the ACCmax lies in a hemodynamically significant stenosis ( ≥ 70% reduction in cross-sectional area). In the present study, there is a relative wide spread in ACCmax at a hemodynamically non-significant stenosis of 50% in cross-sectional area (figure 3(d)), resulting in a non-significant difference between the ACCmax at no stenosis and 50% stenosis. This might be explained by the fact that an acute stenosis was made in young and healthy test subjects. A visual observation of the investigators was that the cardiac output increased (increased stroke volume on DUS-images, however this was not objectified by measurements) as a reaction on the ‘first acute’ stenosis. Therefore, the ACCmax could be increased at some young healthy test subject at a 50% stenosis compared to a non-stenosis. This will probably not occur in patients with PAD since this disease has a more chronic character. Still the ACCmax decreases at a hemodynamically significant stenosis, even in young healthy test subjects. Moreover, in the previous in vitro study, there was a normal decrease in ACCmax between no stenosis and 50% stenosis (diameter reduction) since the ‘cardiac output’ was unchanged during the different test setups.^13^ Despite the relative wide spread at a 50% stenosis in this study, the ACCmax had a higher correlation with the degree of stenosis compared to ABI, TBI, TP and other DUS parameters.

### Limitations

Apart from complex clinical settings that occur in reality (eg impact from cardiac output, shear rate, collateral circulation, vascular compliance and outflow obstruction), this in vivo study investigated basic principles: the impact of different artificial stenosis in healthy subject and compared the ACCmax with conventional non-invasive pressure measurements and DUS parameters. Since the study population consisted of healthy participants, only an artificial single stenosis could be mimicked, while the real PAD patient has often multi-level disease. However, the previous in vitro study revealed a comparable trend with respect to both ACCmax and intra-arterial pressure gradient at multi-level disease.^13^ Furthermore, in the present study, compression of the artery was provided by an ultrasound probe, resulting in an oval shape and smooth surface of the artery, which might distort the results compared to a more rough and irregular arterial stenosis. Note that the degree of stenosis is given in reduction in cross-sectional area as a result of the oval shape of the artificial stenosis. The high ICC in this study might be due to the relatively large intervals in terms of the degree of stenosis (no stenosis, 50%, 70% and 90%). Hence, the high ICC for ACCmax must be interpreted with caution and should be examined in patients in a prospective clinical setting.

## Conclusion

The present study contributes to further evaluation of ACCmax to diagnose and assess the severity of peripheral arterial disease (PAD). The ACCmax correlates more accurately with the degree of stenosis than conventional non-invasive pressure measurements and other DUS parameters in artificially introduced arterial stenosis in healthy individuals. ACCmax measurement can be obtained with a low interobserver variability. Along with the potential benefits of ACCmax concerning MAC, it may provide a reliable new non-invasive technique in PAD. Future investigation in ACCmax is needed in patients with PAD to obtain its exact clinical value and the potential benefits in PAD.
